# Glucosylceramide in cerebrospinal fluid of patients with *GBA*-associated and idiopathic Parkinson’s disease enrolled in PPMI

**DOI:** 10.1038/s41531-021-00241-3

**Published:** 2021-11-22

**Authors:** Young Eun Huh, Hyejung Park, Ming Sum Ruby Chiang, Idil Tuncali, Ganqiang Liu, Joseph J. Locascio, Julia Shirvan, Samantha J. Hutten, Melissa S. Rotunno, Catherine Viel, Lamya S. Shihabuddin, Bing Wang, Sergio Pablo Sardi, Clemens R. Scherzer

**Affiliations:** 1 Center for Advanced Parkinson Research, Harvard Medical School, Brigham & Women’s Hospital, Boston, MA 02115 USA; 2 Precision Neurology Program, Harvard Medical School, Brigham & Women’s Hospital, Boston, MA 02115 USA; 3 Department of Neurology, Brigham and Women’s Hospital, Boston, MA 02115 USA; 4 Department of Neurology, CHA Bundang Medical Center, CHA University, Seongnam-si, Gyeonggi-do 13496 South Korea; 5 NA Pre-Development Sciences, Sanofi, Waltham, MA 02451 USA; 6 Rare and Neurologic Diseases Therapeutic Area, Sanofi, Framingham, MA 01701 USA; 7 School of Medicine, Sun Yat-sen University, Guangzhou, Guangdong 510080 China; 8 Department of Neurology, Massachusetts General Hospital, Boston, MA 02114 USA; 9 The Michael J. Fox Foundation for Parkinson’s Research, New York, NY 10163 USA

**Keywords:** Parkinson's disease, Prognostic markers

## Abstract

Protein-coding variants in the *GBA* gene modulate susceptibility and progression in ~10% of patients with Parkinson’s disease (PD). *GBA* encodes the β-glucocerebrosidase enzyme that hydrolyzes glucosylceramide. We hypothesized that *GBA* mutations will lead to glucosylceramide accumulation in cerebrospinal fluid (CSF). Glucosylceramide, ceramide, sphingomyelin, and lactosylceramide levels were measured by liquid chromatography-tandem mass spectrometry in CSF of 411 participants from the Parkinson’s Progression Markers Initiative (PPMI) cohort, including early stage, de novo PD patients with abnormal dopamine transporter neuroimaging and healthy controls. Forty-four PD patients carried protein-coding *GBA* variants (*GBA*-PD) and 227 carried wild-type alleles (idiopathic PD). The glucosylceramide fraction was increased (*P* = 0.0001), and the sphingomyelin fraction (a downstream metabolite) was reduced (*P* = 0.0001) in CSF of *GBA*-PD patients compared to healthy controls. The ceramide fraction was unchanged, and lactosylceramide was below detection limits. We then used the ratio of glucosylceramide to sphingomyelin (the GlcCer/SM ratio) to explore whether these two sphingolipid fractions altered in *GBA*-PD were useful for stratifying idiopathic PD patients. Idiopathic PD patients in the top quartile of GlcCer/SM ratios at baseline showed a more rapid decline in Montreal Cognitive Assessment scores during longitudinal follow-up compared to those in the lowest quartile with a *P*-value of 0.036. The GlcCer/SM ratio was negatively associated with α-synuclein levels in CSF of PD patients. This study highlights glucosylceramide as a pathway biomarker for *GBA*-PD patients and the GlcCer/SM ratio as a potential stratification tool for clinical trials of idiopathic PD patients. Our sphingolipids data together with the clinical, imaging, omics, and genetic characterization of PPMI will contribute a useful resource for multi-modal biomarkers development.

## Introduction

Mutations and coding variants in the *GBA* gene are found in ~ 7–10% of patients with Parkinson’s disease (PD)^1–3^. *GBA* encodes the β-glucocerebrosidase enzyme that hydrolyzes the substrate glucosylceramide. In PD patients, increasing severity of the type of *GBA* mutation is quantitatively associated with decreasing β-glucocerebrosidase activity^4^, increased risk of developing PD^5^, and more rapid cognitive decline^2,6^. We and others^6^ previously reported that neuropathic Gaucher’s disease (GD) mutations are linked to rapid cognitive decline in PD patients^2,6–8^. The strongest effect was seen for severe, neuropathic GD mutations^2,6^. In GD patients, homozygous *GBA* mutations lead to dramatic glucosylceramide accumulation in brain and body fluids^9–12^. Medications that lower glucosylceramide by replacing the deficient enzyme or through inhibition of glucosylceramide synthesis are highly effective treatments for GD. However, current commercially-available enzyme replacing or substrate reducing therapeutics are limited in their efficacy for neuropathic GD patients due to their inability to penetrate the blood brain barrier^13^.

The precise mechanism through which *GBA* mutations contribute to the pathobiology of PD, a common neurodegenerative movement disorder, is unclear^14^. One hypothesis is that these mutations cause a loss of enzyme function leading to the accumulation of the substrate glucosylceramide, the direct substrate of β-glucocerebrosidase. Glucosylceramide is the sphingolipid component of cell membranes and consists of sphingosine, a fatty acid chain (these two forming a ceramide), and a glucose moiety^15^. Glucosylceramide is synthesized in the Golgi apparatus by glucosylceramide synthase via the transfer of a glucose residue from UDP-glucose to ceramide. It is found in all mammalian tissues, particularly abundant in the brain, and is required for intracellular membrane trafficking, signal activity, and cell proliferation^15^. Excessive glucosylceramide may promote the formation of toxic species of α-synuclein by converting physiological α-synuclein conformers into stable, assembly-state intermediates^16^. Accumulated α-synuclein blocks the endoplasmic reticulum-Golgi trafficking of the β-glucocerebrosidase, thus further exacerbating lysosomal dysfunction and glucosylceramide accumulation^14,17^. Substrate-reducing therapeutics that cross the blood-brain barrier are needed, and several such compounds are at pre-clinical and clinical stages of development. However, substrate accumulation has thus far not been demonstrated in *GBA*-PD patients, possibly due to the lack of assays specific for glucosylceramide, small sample sizes, and potential confounding from dopamine replacement medications.

Based on this pathogenetic model, we hypothesized that glucosylceramide levels should be increased in cerebrospinal fluid (CSF) of PD patients with *GBA* mutations (*GBA*-PD). Here we tested this question using a quantitative analysis of the sphingolipids method based on liquid chromatography with tandem mass spectrometry analysis (LC/MS/MS). CSF was obtained from patients enrolled in the Michael J. Fox Foundation’s Parkinson’s Progression Markers Initiative (PPMI) cohort^18^, a large, well-phenotyped collection of early-stage, neuroimaging-confirmed patients with PD and controls.

## Results

### Clinical baseline characteristics

The clinical characteristics of the 411 participants are presented in Table 1. *GBA*-PD patients were younger than idiopathic PD patients (*P* = 0.005) and healthy controls (*P* = 0.043). *GBA*-PD patients had an earlier age at onset compared to idiopathic PD patients. Montreal Cognitive Assessment (MoCA) scores in the groups of *GBA*-PD patients (*P* = 0.021) and idiopathic PD patients (*P* < 0.001), respectively, were statistically lower than in the healthy control group (although the median MoCA score was 28 in both the healthy controls and the *GBA*-PD patients). MoCA scores and the Movement Disorder Society-Unified Parkinson’s Disease Rating Scale (MDS-UPDRS) part III scores at baseline were similar in *GBA*-PD patients and idiopathic PD patients. Sex, disease duration at baseline, years of education, and body mass index (BMI) were similar in the three groups of participants.

**Table 1:** Clinical characteristics and *GBA* genotypes of participants from the Parkinson’s Progression Markers Initiative (*n* = 411).

	Healthy controls (*n* = 140)	Idiopathic PD (*n* = 227)	*GBA*-PD (*n* = 44)	*P*-value
Age, y	63.0 (56.0–70.0)	65.0 (57.0–70.0)	60.5 (54.8–65.0)	0.019*‡
Male, n (%)	92 (65.7%)	148 (65.2%)	26 (59.1%)	0.707
Age at onset, y	NA	63.0 (56.0–69.0)	59.0 (54.0–64.0)	0.006*
Disease duration, y	NA	1.0 (0.0–1.0)	1.0 (0.0–1.0)	0.924
MDS-UPDRS III	0.0 (0.0–2.0)	20.0 (15.0–27.0)	23.0 (15.5–28.0)	<0.001†‡
MoCA	28.0 (27.0–29.0)	27.0 (26.0–29.0)	28.0 (26.0–29.0)	<0.001†‡
Years of education	16.0 (14.0–18.0)	16.0 (14.0–18.0)	16.0 (14.0–18.0)	0.164
BMI, kg/m^2^	26.5 (24.1–29.6)	26.5 (24.0–30.4)	26.0 (24.2–29.2)	0.784
*GBA* genotypes (n)	NA	NA	*GBA* risk variants (29): E326K (19), T369M (10) Mild *GBA* mutations (7): N370S (7) Severe *GBA* mutations (8): L444P (2), A456P (2), R463C (1), IVS2 + 1 G > A (1), T369M/R120W (1), N370S/N370S (1)	

Values are median (interquartile range) unless otherwise stated.

Kruskal–Wallis, Mann–Whitney, or *χ*^2^ tests were used as appropriate.

*P < 0.05 *GBA*-PD vs idiopathic PD, †*P* < 0.05 idiopathic PD vs HC, ‡ *P* < 0.05 *GBA*-PD vs HC.

BMI body mass index, GBA-PD Parkinson disease patients with *a GBA* mutation, Idiopathic PD Parkinson disease patients without *GBA* mutation, MoCA Montreal Cognitive Assessment, MDS-UPDRS III Movement Disorder Society-Unified Parkinson Disease Rating Scale part III.

### Cross-sectional sphingolipids analyses of *GBA*-PD, idiopathic PD, and controls

**Fig. 1:**
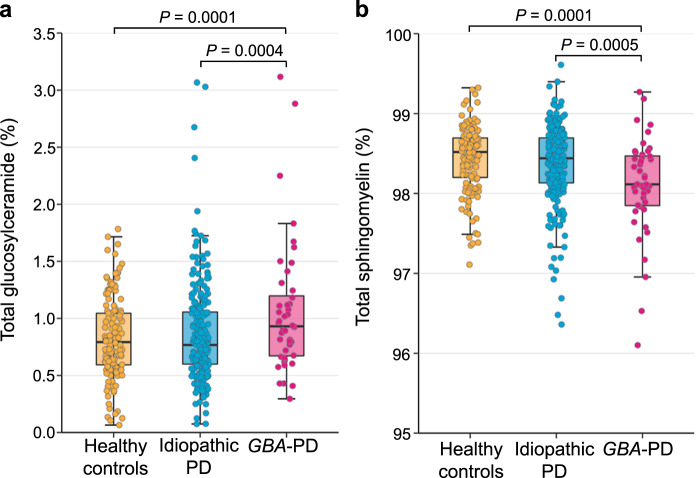
Glucosylceramide and sphingomyelin in cerebrospinal fluid (CSF) of *GBA*-PD patients compared to idiopathic PD patients and healthy controls. **a** The glucosylceramide fraction was significantly higher in *GBA*-PD patients compared to idiopathic PD patients and healthy controls adjusting for covariates. **b** The sphingomyelin fraction was significantly lower in *GBA*-PD patients compared to idiopathic PD patients and healthy controls controlling for covariates. Unadjusted sphingolipid fractions in CSF are shown in box and jitter dot blots; box plots indicate the median (bold line), the 25th and 75th percentiles (box edges), and the most extreme data point no more than 1.5x the interquartile range from the box (whiskers). The *P*-values are from linear mixed model analyses adjusting for covariates.

**Fig. 2:**
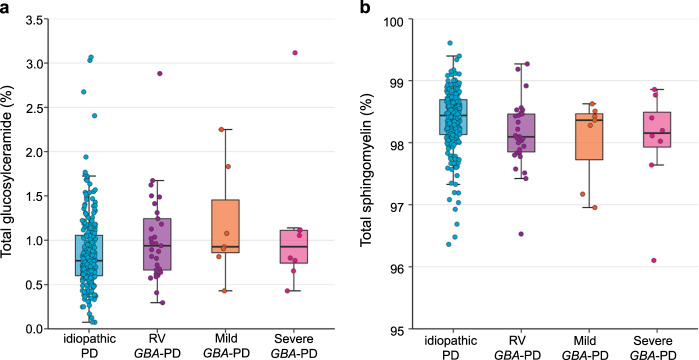
Glucosylceramide and sphingomyelin in PD patients with distinct types of *GBA* variants. **a** Glucosylceramide fraction in patients with idiopathic PD and in *GBA*-PD patients carrying risk variants (RV), mild mutations, and severe mutations. **b** Sphingomyelin fraction in patients with idiopathic PD and in *GBA*-PD patients with risk variants (RV), mild mutations, and severe mutations. Unadjusted sphingolipid fractions in CSF of 271 PD patients are shown in box and jitter dot blots.

The glucosylceramide fraction was significantly increased (Fig. 1a) and the sphingomyelin fraction was significantly reduced (Fig. 1b) in *GBA*-PD patients adjusted for covariates, respectively. The median glucosylceramide fraction in *GBA*-PD patients was 0.93% (IQR, 0.67–1.20) compared to 0.79% (0.59–1.04) in healthy controls (*P* = 0.0001). The median sphingomyelin fraction in *GBA*-PD patients was 98.11% (97.85–98.47) compared to 98.52% (98.20–98.69) in healthy controls (*P* = 0.0001). Levels in idiopathic PD patients were similar to those in healthy controls for the glucosylceramide fraction (0.77%, 0.60–1.06) and for the sphingomyelin fraction (98.44%, 98.13–98.70). Glucosylceramide and sphingomyelin fractions in PD patients with distinct types of *GBA* genotypes, e.g., wild-type *GBA* allele, *GBA* risk variants, mild mutations, and severe *GBA* mutations (see “Methods” sections for detail), are shown in Fig. 2. The ceramide fractions in healthy controls (0.70%, 0.58–0.84), idiopathic PD patients (0.73%, 0.60–0.87), *GBA*-PD patients (0.80%, 0.65–1.00) did not statistically significantly differ. Lactosylceramide was below detection limits.

### Longitudinal sphingolipids analysis of *GBA*-PD, idiopathic PD, and controls

For longitudinal analysis, a subset of 341 participants was available for analysis, including 38 *GBA*-PD patients, 189 idiopathic PD patients, and 114 healthy controls. The median follow-up duration was 3.0 years (IQR, 2–4 years; maximum, 3.5 years). Baseline characteristics are shown in Supplementary Table 1. After backwards elimination, linear mixed model analysis indicated a significant main effect of age at baseline on the glucosylceramide fraction (*P* = 0.0064; higher in older participants) and of sex on the ceramide fraction (*P* = 0.0002; lower in males) (Supplementary Table 2). The group differences of elevated glucosylceramide fraction and reduced sphingomyelin fraction in *GBA*-PD patients compared to healthy controls observed at baseline remained stable and significant over time with *P*-values of 0.010 and 0.006, respectively in the longitudinal analysis. There was no appreciable difference in the slope of the glucosylceramide fraction (*P* = 0.120 for *GBA*-PD; *P* = 0.292 for idiopathic PD), ceramide fraction (*P* = 0.394; *P* = 0.694), and sphingomyelin fraction (*P* = 0.111; *P* = 0.587) over time either in *GBA*-PD patients or in idiopathic PD patients compared to healthy controls. That is, there was no significant interaction between group and time in study.

There was no significant association between longitudinal sphingolipid fractions and longitudinal MoCA scores using linear mixed model analysis adjusted for covariates (*P* = 0.475 for the glucosylceramide fraction; *P* = 0.205 for the sphingomyelin fraction; *P* = 0.220 for the ceramide fraction). Similarly, there were no significant associations between longitudinal sphingolipids fractions and longitudinal MDS-III scores (*P* = 0.074 for the glucosylceramide fraction; *P* = 0.161 for the sphingomyelin fraction; *P* = 0.783 for the ceramide fraction).

Thus, baseline glucosylceramide and sphingomyelin profiles are abnormal in CSF of early-stage, de novo *GBA*-associated PD without appreciable longitudinal changes during the follow-up period captured in PPMI.

### Stratification of idiopathic PD based on the ratio of glucosylceramide to sphingomyelin (GlcCer/SM ratio)

**Fig. 3:**
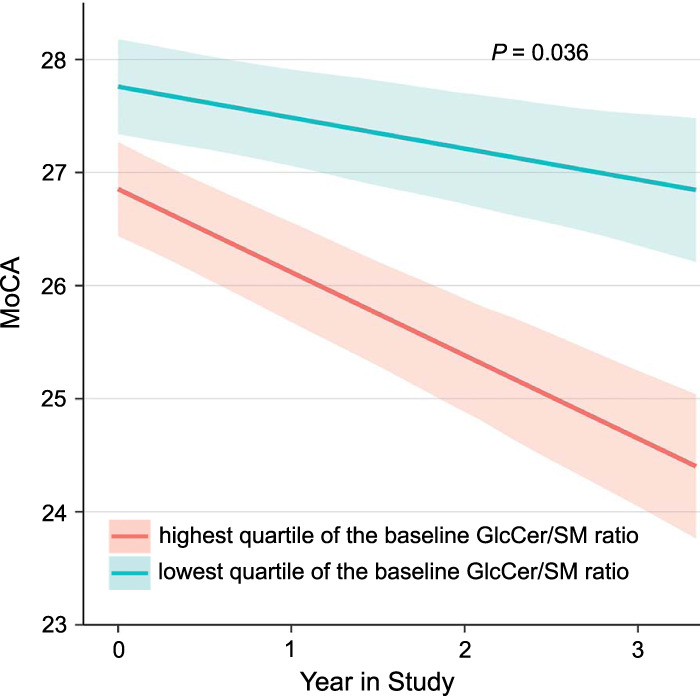
Stratification of idiopathic PD based on the ratio of glucosylceramide to sphingomyelin (GlcCer/SM ratio) at enrollment. Idiopathic PD patients with the highest quartile of GlcCer/SM ratio at enrollment (magenta) had an accelerated longitudinal cognitive decline compared to those with the lowest quartile of GlcCer/SM ratio (cyan). Model-predicted mean Montreal Cognitive Assessment (MoCA) scores (solid lines) and standard errors (shaded areas) across time are shown for idiopathic PD patients in the highest (*n* = 48) and lowest (*n* = 47) quartile of GlcCer/SM ratios at enrollment visits. Fixed effects covariates included in the model were time in study (years), GlcCer/SM ratio, the interaction of GlcCer/SM ratio and time in study, age at baseline, the interaction of age at baseline and time in study, and sex. For the purpose of the graph, sex was arbitrarily set to female. Similar results were seen when sex was set to male (not shown).

We then sought to explore whether this *GBA* mutation-linked sphingolipids profile (characterized by an elevated glucosylceramide fraction and a reduced sphingomyelin fraction) is useful for stratifying common, idiopathic PD patients *without* known *GBA* mutations. To that end we stratified the patients with de novo, idiopathic PD in PPMI based on their ratio of glucosylceramide to sphingomyelin (GlcCer/SM ratio) measured at baseline. We then asked whether patients in the highest quartile of the GlcCer/SM ratio had a more rapid cognitive disease progression compared to those patients within the lowest quartile of the GlcCer/SM ratio. We compared longitudinal MoCA scores of idiopathic PD patients in the highest quartile of GlcCer/SM ratio at baseline to those in the lowest quartile of GlcCer/SM ratio using a linear mixed effect model. Importantly, baseline clinical characteristics did not differ between patients in the top quartile of GlcCer/SM ratio and those in the lowest (reference) quartile (Supplementary Table 3). We found that idiopathic PD patients in the highest quartile of a GlcCer/SM ratio had an accelerated decline in MoCA scores over time compared to those in the bottom quartile of a GlcCer/SM ratio (Fig. 3, Supplementary Table 4) with *P* = 0.036. We also compared longitudinal MoCA scores of *GBA*-PD patients in the highest quartile of the GlcCer/SM ratio at baseline to those in the lowest quartile of the GlcCer/SM ratio using a linear mixed effect model. Age at baseline (*P* = 0.008) and age at onset (*P* = 0.008) were significantly younger in *GBA*-PD patients in the highest quartile of GlcCer/SM ratio at baseline compared to those in the lowest quartile (Supplementary Table 5). There was no difference in MoCA scores, MDS-UPDRS III scores, years of education, and BMI at baseline between *GBA*-PD patients in the highest quartile of the baseline GlcCer/SM ratio and those in the lowest quartile. We found the MoCA scores more rapidly decreased in *GBA*-PD patients in the highest quartile compared to those in the lowest quartile, similar to idiopathic PD patients (*P* = 0.029) (Supplementary Table 6). However, the significance of the effect of the baseline GlcCer/SM ratio on the cognitive outcome in *GBA*-PD patients should be interpreted cautiously due to the small number of patients included in each quartile (*n* = 10). There were no significant associations between the baseline GlcCer/SM ratio and the longitudinal MDS-UPDRS III scores in idiopathic PD patients (*P* = 0.210) and in *GBA*-PD patients (*P* = 0.733), respectively.

### Association between the α-synuclein levels and glucosylceramide fraction in CSF of PD patients

The level of α-synuclein in CSF was decreased in *GBA*-PD (median = 1334.10 pg/mL, IQR = 1064.97–1713.45, *P* = 0.005) and idiopathic PD patients (1435.00 pg/mL, 1088.95–1875.50, *P* = 0.001) compared to healthy controls (1686.50 pg/mL, 1236.77–2233.20). The α-synuclein level did not differ significantly between *GBA*-PD and idiopathic PD patients. We evaluated whether the glucosylceramide fraction was associated with α-synuclein levels in CSF of PD patients using a multivariable linear regression analysis. After a limited backward elimination, the model showed a significant effect of age at onset (*P* = 0.005; higher in patients with older age at onset) and sex (*P* = 0.049; lower in male patients) on the level of α-synuclein in CSF of PD patients. The glucosylceramide fraction appeared negatively associated with the level of α-synuclein in CSF of PD patients after adjusting for the covariates of age at onset and BMI (*P* = 0.041). We also used a similar statistical analysis to assess the association between the level of α-synuclein and the GlcCer/SM ratio in CSF and obtained consistent results: that is, a significant negative association between the level of α-synuclein and the GlcCer/SM ratio in CSF of PD patients (*P* = 0.040).

## Discussion

This study shows a perturbation of the sphingolipids pathway detectable in CSF of patients with early-stage, neuroimaging-supported, de novo PD carrying a *GBA* mutation. Consistent with our hypothesis *GBA* mutations led to a statistically significant glucosylceramide accumulation in CSF. The relative abundance of the direct substrate of the β-glucocerebrosidase enzyme, glucosylceramide, was significantly increased and the sphingomyelin fraction (a key downstream metabolite) was reduced in CSF of *GBA*-PD patients. Interestingly, the glucosylceramide/sphingomyelin signature derived from *GBA*-associated genetic forms of PD might be transferable to stratifying idiopathic PD patients. In idiopathic PD patients, a higher ratio of CSF glucosylceramide to CSF sphingomyelin (GlcCer/SM ratio) assayed at enrollment was associated with a more rapid longitudinal cognitive decline compared to patients with a lower GlcCer/SM ratio. This suggests that a subset of idiopathic PD patients has biochemical pathway changes that mimic those seen in *GBA*-related PD. Thus, idiopathic PD patients with a high GlcCer/SM ratio may be candidates for targeted therapies designed to reduce the glucosylceramide substrate.

These data build on recent work from multiple large, well-powered, and deeply phenotyped biomarkers cohorts^2–4^. In these PD cohorts, *GBA* mutations are linked to early disease onset and rapid progression^2^. Biochemically, PD patients carrying *GBA* variants have reduced β-glucocerebrosidase enzyme activity in dried blood spots^4,19^. Taken together, these converging lines of evidence are consistent with the hypothesis that *GBA* mutations lead to a partial loss of β-glucocerebrosidase activity and substrate accumulation in patients with PD.

The mechanistic interactions between glucosylceramide and α-synuclein aggregation are under intense investigation^14^. α-Synuclein-lipid interactions have long been thought to play an important role in modulating α-synuclein aggregation^20^. In patients with GD, homozygous loss-of-function mutations in the *GBA* gene result in a reduction in β-glucocerebrosidase activity and an accumulation of glucosylceramide, and deposition of α-synuclein-positive Lewy bodies. In *GBA* mutant mice carrying the human A30P-α-synuclein transgene, glucosylsphingosine is accumulated in young mice and, with aging, brains accumulate glucosylceramide colocalized with α-synuclein pathology^21^. GD-related glycosphingolipids (e.g., glucosylceramide, glucosylsphingosine) promote wild-type α-synuclein aggregation into β**-**sheeted conformation in vitro based on circular dichroism studies^21^. Glycosphingolipids also increase the conversion of physiological α-synuclein conformers into toxic α-synuclein aggregates in induced pluripotent stem cell (iPSC)-derived midbrain dopamine neurons from GD patients or those from healthy controls treated with β-glucocerebrosidase inhibitor, conduritol-b-epoxide^16^. By contrast, glycosphingolipid-lowering compounds (e.g., glucosylceramide synthase inhibitor) appear to reverse the formation of pathological α-synuclein aggregates in cell lines and neurons from patient-derived induced pluripotent stem cells^16^ and animal models of synucleinopathy^22^. Additionally, glucosylceramide accumulation itself can be toxic to neurons^23^. In line with experimental data, we found a significant association between glucosylceramide fraction and α-synuclein in CSF of PD patients. Previous studies have reported decreased level of CSF α-synuclein in PD patients^24–26^. This is thought to reflect reduced release of α-synuclein into CSF due to α-synuclein aggregation in Lewy bodies in the brain of PD patients. This is analogous to the observation of low CSF Aβ_1–42_ levels in the brain of AD patients. Accordingly, the association between high GlcCer/SM and low α-synuclein level in CSF observed in this study is consistent with the hypothesis of increased α-synuclein accumulation induced by sphingolipid alterations in the brain of PD patients. Furthermore, the association between the GlcCer/SM ratio and α-synuclein could potentially also partly explain the association between baseline GlcCer/SM ratio and cognitive prognosis as other studies reported that decreased CSF α-synuclein levels were related to cognitive decline in drug-naive early-stage PD patients^27,28^.

While the data based on peripheral biofluids are becoming increasingly robust based on the analyses of hundreds of individuals, the pathway changes in human neuronal tissues of carriers of *GBA* mutations remain, in part, unclear. Our study did not directly evaluate sphingolipids in human brain autopsies, which is not possible in PPMI as most participants are still alive. In *GBA*-PD patients, a moderate reduction in β-glucocerebrosidase activity has been previously found in postmortem brain tissue^29^, CSF^30^, and peripheral blood^4,19^. Moreover, in human dopamine neurons induced from pluripotent patient stem cells with heterozygous *GBA* mutations, β-glucocerebrosidase activity is reduced and glucosylceramide levels are increased^31^, consistent with the clinical observations in CSF from the PPMI cohort. Substrate accumulation, including glucosylsphingosine and galactosylsphingosine, was also reported in dry blood spots from *GBA*-PD patients^32^. Altered levels of ceramide and sphingomyelin were documented in the serum samples of *GBA*-PD patients^33^. Two pilot studies of brain autopsy samples from *GBA*-PD patients, however, either found no increase in glucosylceramide^34^ or a non-significant trend towards an increase in glucosylceramide^35^, respectively. Studies based on human brain autopsies offer the most direct window into the neuropathobiology of *GBA*-PD, but are limited by sample sizes, processing parameters (e.g., post-mortem interval, brain tissue quality), antemortem medication treatment and comorbidity. Moreover, these autopsy brain studies have examined brain homogenates, which are a heterogeneous mix of multiple cell types that can obscure cell type-specific effects (e.g., neuronal vs. glial changes). Thus, quantifying small changes in glucosylceramide and other *GBA* pathway metabolites in heterozygous carriers of *GBA* mutations is challenging and requires sensitive methods, standardized biospecimens processing procedures, and large sample sizes that provide the statistical power necessary to detect the modest changes expected for this chronic disease. Cell type-specific investigations of cortex and substantia nigra of well-powered cohorts of human PD brains will be needed to conclusively address the neuronal effects of heterozygous *GBA* variants.

This study also has limitations. Firstly, while overall a substantial number of *GBA*-PD patients was analyzed, the sub-group analyses were limited and require further evaluation. Secondly, to confidently assess for associations (or lack thereof) between CSF sphingolipids levels and clinical phenotypes larger sample sizes might be required. Clinical scales are highly variable due to inter-individual and inter-rater variability. This variation complicates correlations between clinical assessments and sphingolipids. In prior work more than two thousand PD patients (including 198 *GBA*-PD patients) with 20,868 longitudinal study visits^2^ were needed to uncover a significant link between *GBA* mutations and longitudinal decline in clinical cognitive assessments due to variation in clinical assessments. Previously, we did not detect longitudinal correlations between longitudinal β-glucocerebrosidase activity and cognitive or motor scores in 195 participants from the HBS and PDBP cohorts^4^, similar to the current study. In this study, we used the relative abundance (or fraction) of sphingolipids and introduced the ratio of glucosylceramide to sphingomyelin. These relative measures may capture more information in terms of changes in the pertinent biological process and normalize for differences in substrate loading. Finally, our study identified an association between *GBA* genotype and CSF sphingolipids levels in PD, but did not address whether the *GBA* genotype actually *causes* the clinical phenotype via quantitative modulation of glucosylceramide levels.

In summary, this study links heterozygous *GBA* variants to an abnormal sphingolipids profile in CSF of PD patients that is consistent with the loss-of-function hypothesis. It highlights a potential CSF biomarker for stratifying idiopathic PD patients in clinical trials. Precision medicine requires a deep understanding of PD that integrates data across genetics, omics, imaging, and clinical phenotypes, and dynamically traces the behavior of these multi-scale systems across time. The sphingolipids data here generated add to the rich molecular, imaging, and clinical characterization of the PPMI cohort. This openly accessible metabolic characterization of a core genetic pathway of PD will be a useful resource for multi-modal dissection of the complex pathobiology of PD.

## Methods

### Study design and participants

CSF samples, clinical and genetic data used in the preparation of this article were obtained from the Parkinson’s Progression Markers Initiative (PPMI) database (www.ppmi-info.org/data)^18^. For up-to-date information on the study, visit www.ppmi- info.org. Enrollment criteria for PD participants in PPMI were age older than 30 years old; diagnosis of PD within 2 years prior to the screening visit; asymmetric resting tremor or asymmetric bradykinesia; or two of the following: bradykinesia, rigidity, and resting tremor; untreated for PD at the baseline visit; be in Hoehn and Yahr stage 1 or 2; and have a dopamine transporter deficit on imaging^18^. Motor disability and global cognitive function were assessed at each study visit by using the Movement Disorder Society-Unified Parkinson’s Disease Rating Scale (MDS-UPDRS) part III and Montreal Cognitive Assessment (MoCA), respectively, from baseline to 3-year follow-up. Detailed protocols for participants selection, clinical assessment, and data collection in PPMI have been described previously^18^.

*GBA* genotyping was performed for PPMI by Dr. Andy Singleton at the National Institutes of Health and curated *GBA* genotypes were provided for this study by Dr. Roy Alcalay of Columbia University. Individuals with a known *LRRK2* G2019S mutation were excluded for this analysis. *GBA* mutations of unknown significance, including G105R/G183E, I479L, R78C, R83C, were also excluded. A total of 411 participants were analyzed, including 44 *GBA*-PD patients, 227 idiopathic PD patients, and 140 healthy controls. Longitudinal analysis was conducted using a subset of 341 participants with CSF sphingolipid profiles available from baseline and at least one follow-up time point, including 38 *GBA*-PD patients, 189 idiopathic PD patients, and 114 healthy controls (for the flow chart of the study participants see “Supplementary Fig. 1”). The median follow-up duration was 3.0 years (IQR, 2–4 years; maximum, 3.5 years) and the number of visits ranged from 2 to 4.

The present analysis of de-identified data from PPMI was approved by the Institutional review board of Brigham and Women’s Hospital. PPMI was approved by the ethics committees at each participating site, and written informed consent was obtained from all participants prior to inclusion in the study.

### Sphingolipid extraction from human CSF

Ceramide in human CSF was extracted by protein precipitation. Briefly, 30 μL of human CSF were suspended in 60 μL methanol and 30 μL chloroform. Methanol contained 1 ng/mL of C12:0 Ceramide as an internal standard (IS) (Avanti polar lipids, Alabaster, AL). After vortexing and centrifugation, the supernatant was dried with N_2_ gas and resuspended in 120 μL of solvent (acetonitrile/methanol, 90/10, v/v) for ceramide analysis. Glucosylceramide and lactosylceramide in human CSF were extracted by liquid–liquid extraction. Fifty microliters of human CSF were suspended in 1 mL methanol, 1.5 mL chloroform, and 2 mL water. Methanol contained 0.25 ng/mL of C12:0 glucosylceramide, and 1.25 ng/mL of C12:0 lactosylceramide as IS (Avanti polar lipids, Alabaster, AL). After vortexing and centrifugation, the lower phase was carefully transferred and dried using N_2_ gas and redissolved in 0.2 mL of solvent (acetonitrile/methanol, 90/10, v/v) for glucosylceramide and lactosylceramide analysis. For sphingomyelin analysis, 10 μL of human CSF were extracted by protein precipitation in extraction solution (acetonitrile/methanol/acetic acid/water, 96/2/1/1, v/v/v/v, with 5 mM ammonium acetate) using Hamilton STAR liquid handler (Reno, NV). The extraction solvent contained 5 ng/mL of C12:0 sphingomyelin (Avanti polar lipids, Alabaster, AL) as an IS. The mixtures were vortexed and centrifuged. The resulting supernatants were transferred to 384-well plates for sphingomyelin analysis.

### Quantitative analysis of sphingolipids

Quantitative analysis of sphingolipids was performed using liquid chromatography with tandem mass spectrometry (LC-MS/MS). Ceramide, glucosylceramide, and lactosylceramide were separated using a waters 2.1 × 100 mm Cortecs HILIC column at a flow rate of 0.5 mL/min and a Waters Acquity binary solvent manager (Waters Corporation, Milford, MA). Mobile phase A consisted of acetonitrile/methanol/acetic acid/water = 96/2/1/1, v/v/v/v, and 5 mM ammonium acetate. Mobile phase B consisted of methanol/water/acetic acid = 79/20/1, v/v/v, and 5 mM ammonium acetate. The LC eluents were analyzed by a triple quadrupole mass spectrometer (AB Sciex API 5000, Foster City, CA) in MRM mode. Calibration curves were generated with Ceramide of C16:0, C18:0, C20:0, C22:0, C24:1, and C24:0 (Avanti polar lipids, Alabaster, AL), lactosylceramide of C16:0, C18:0, C24:1, and C24:0 (Avanti polar lipids, Alabaster, AL), and glucosylceramide mixture standard (Matreya, LLC, State College, PA). Representative results of MS data for glucosylceramide, ceramide, and lactosylceramide are presented in Supplementary Fig. 2. Sphingomyelin was separated from phosphatidylcholines (PC) using a Waters 2.1 × 100 mm Cortecs HILIC column at a flow rate of 0.5 mL/min and a Waters Acquity binary solvent manager (Waters Corporation, Milford, MA). The LC eluents were analyzed by a triple quadrupole mass spectrometer (AB Sciex API 4000, Foster City, CA) in MRM mode. Calibration curves were generated with sphingomyelin of C16:0, C18:0, C24:1, and C24:0 (Avanti polar lipids, Alabaster, AL) and C20:0 and C22:0 (Matreya, LLC, State College, PA).

We calculated the fraction (%) of glucosylceramide, ceramide, and sphingomyelin out of the total CSF sphingolipids by dividing the abundance of each sphingolipid (ng/mL) by the total abundance (ng/mL) of the three input sphingolipids^36^.

### A priori operational classification of *GBA* mutation type

To explore sphingolipids levels in patients with different types of *GBA* mutations, *GBA*-PD patients were divided into three operational subgroups based on the historic association of mutations with GD with central nervous system (CNS) involvement (“neuropathic”) or without CNS involvement (“non-neuropathic”) as previously published^2,4^., Briefly, (1) **PD carriers of severe** ***GBA*** **mutations**. This group includes PD patients with *GBA* mutations that are associated with neuropathic GD, including L444P, L444R, A456P, and R120W. These *GBA* mutations are reported to markedly increase the risk for PD and accelerate cognitive impairment in PD patients^2^. PD patients carrying complex *GBA* alleles (e.g., homozygotes carriers of severe or mild *GBA* mutations or homozygous PD-associated *GBA* risk variants; or compound heterozygotes carriers) were also a priori assigned to this group, as these patients showed aggressive cognitive deterioration similar to PD patients with *GBA* mutations linked to neuropathic GD in prior work^2^. (2) **PD carriers of mild** ***GBA*** **mutations**. This group includes PD patients with *GBA* mutations, such as N370S that cause non-neuropathic GD. The disease risk and the rate of cognitive decline may be moderately elevated in this group in some studies^6^. (3) **PD-associated** ***GBA*** **risk variants**. This group includes PD patients with PD-associated, protein-coding *GBA* variants (E326K, T369M, and E388K). These variants have been associated with increased risk for PD, earlier disease onset, and progression of motor and cognitive impairment in multiple studies^37,38^; however, they are not per se pathogenic for GD^39^. (4) **Idiopathic PD patients** were defined for this study as PD patients without known *GBA* or G2019S *LRRK2* mutations. (5) Healthy controls without a known *GBA* variant or G2019S *LRRK2* mutations were termed “**healthy controls**”.

### Statistical analysis

To compare baseline clinical characteristics between *GBA*-PD patients, idiopathic PD patients, and healthy controls, the Kruskal–Wallis or Mann–Whitney test was used for continuous variables, and the *χ*^2^ test was used for categorical variables.

To examine the effect of *GBA* mutations on sphingolipid fractions, we used a linear mixed effects model for the cross-sectional analysis in 44 *GBA*-PD patients, 227 idiopathic PD patients, and 140 healthy controls. Age, sex, duration of sample storage at baseline, and body mass index (BMI) were entered into the model as fixed covariates. Assay plate was included in the model as a random effect.

To examine the effect of *GBA* mutations on the longitudinal change in sphingolipid fractions, we performed linear mixed effects model analysis. The dependent variable was the sphingolipid fraction. Fixed predictors were time in study (years), group (*GBA*-PD, idiopathic PD, healthy controls), sex, age at baseline, duration of disease at baseline (set to zero for healthy controls), sample storage time at baseline, and the interactions of time in study with the group, age at baseline, sex, or sample storage time at baseline. A random intercept and slope for the effect of time per subject and assay plate were included as random terms.

To evaluate whether the GlcCer/SM ratio measured at enrollment can predict cognitive prognosis longitudinally in PD, we performed a general linear mixed effects model analysis in idiopathic PD patients (e.g., without known *GBA* variants or *LRRK2* mutations) with at least one longitudinal follow-up MoCA exam in addition to the baseline MoCA. Idiopathic PD patients were grouped based on each quartile of the GlcCer/SM ratio at baseline. Longitudinal decline in MoCA scores was compared between patients in the highest quartile of baseline GlcCer/SM ratio and those in the lowest quartile of baseline GlcCer/SM ratio. The dependent variable was MoCA scores and fixed predictors were time in study, group (idiopathic PD patient in the highest quartile of baseline GlcCer/SM ratio and those in the lowest quartile), an interaction between time in study and group, age at baseline, sex, duration of disease at baseline, age at onset, duration of education (year), and the interactions of time in study with age at baseline, sex, or duration at baseline. A random intercept and slope for the effect of time per subject were entered into the model as a random effect. We also evaluated whether the baseline GlcCer/SM ratio can predict the longitudinal cognitive outcome in *GBA*-PD patients using a similar statistical analysis.

We also assessed whether the baseline GlcCer/SM ratio is associated with longitudinal motor outcome in idiopathic PD patients or in *GBA*-PD patients using linear mixed effects model analysis. The dependent variable was MDS-UPDRS III scores and fixed predictors were time in study, group (idiopathic PD patients or *GBA*-PD patients) in the highest quartile of the baseline GlcCer/SM ratio and those in the lowest quartile, an interaction between time in study and group, age at baseline, sex, duration of disease at baseline, age at onset, BMI, and the interactions of time in study with age at baseline, sex, or duration at baseline.

To assess the association between glucosylceramide fraction and the level of α-synuclein in CSF, we used multivariable linear regression analysis with a primary predictor of glucosylceramide fraction in CSF of PD patients. Covariates were age, sex, *GBA* mutation status (presence vs. absence), and interaction between *GBA* mutation status and glucosylceramide fraction. Variables with *P* < 0.2 from the Pearson correlation were also included as covariates, such as age at onset and BMI.

A limited backward elimination procedure was employed to test and remove non-significant variables and higher-order terms that were not of primary substantive interest. *P* < 0.05 was considered nominally significant. *P* < 0.017 (i.e., 0.05/number of tests = 0.05/3) were considered statistically significant after Bonferroni adjustment for multiple testing. Statistical analysis was performed using R (version 3.5.2). The pairwise deletion method was implemented for missing values.

### Reporting Summary

Further information on research design is available in the Nature Research Reporting Summary linked to this article.

## Supplementary information

Supplementary Information

Reporting Summary

## Data Availability

The clinical and lipidomics datasets analyzed during and/or generated during the current study are publicly available in the PPMI repository (www.ppmi-info.org/access-data-specimens/download-data).

## Code availability

We used lme4 package (version 1.1–21) for the linear mixed effects model analysis.
