# Supplementary material for: Glucosylceramide in cerebrospinal fluid of patients with GBA-associated and idiopathic Parkinson’s disease enrolled in PPMI
Source: NPJ Parkinsons Dis. 2021 Nov 22;7:102. doi: 10.1038/s41531-021-00241-3 (PMC8608962; doi:10.1038/s41531-021-00241-3)
Supplement: Supplementary file 1 — Supplementary Information [file 41531_2021_241_MOESM1_ESM.pdf]

Supplementary information

Glucosylceramide in Cerebrospinal Fluid of Patients with *GBA*-associated and Idiopathic Parkinson's Disease enrolled in PPMI

Young Eun Huh[1,2,3,4], Hyejung Park[5], Ming Sum Ruby Chiang[7], Idil Tuncali[1,2,3], Ganqiang Liu[1,2,3,6], Joseph J. Locascio[1,2,3,8], Julia Shirvan[1,8], Samantha J. Hutten[9], Melissa S. Rotunno[7], Catherine Viel[7], Lamya S. Shihabuddin[7], Bing Wang[5], Sergio Pablo Sardi[7] and Clemens R. Scherzer[1,2,3,8]

[1]Center for Advanced Parkinson Research, Harvard Medical School, Brigham & Women's Hospital, Boston, MA 02115, USA

[2]Precision Neurology Program, Harvard Medical School, Brigham & Women's Hospital, Boston, MA 02115, USA

[3]Department of Neurology, Brigham and Women's Hospital, Boston, MA 02115, USA

[4]Department of Neurology, CHA Bundang Medical Center, CHA University, Seongnam-si, Gyeonggi-do, 13496, South Korea

[5]NA Pre-Development Sciences, Sanofi, Waltham, MA 02451, USA

[6]School of Medicine, Sun Yat-sen University, Guangzhou, Guangdong, 510080, China

[7]Rare and Neurologic Diseases Therapeutic Area, Sanofi, Framingham, MA 01701, USA

[8]Department of Neurology, Massachusetts General Hospital, Boston, MA 02114, USA

[9]The Michael J. Fox Foundation for Parkinson's Research, New York, NY 10163, USA

*Correspondence should be addressed to:

Clemens Scherzer
Center for Advanced Parkinson Research
Harvard Medical School, Brigham & Women's Hospital
Hale Building for Transformative Medicine, Laboratory 9002V
Boston, MA 02115
Phone: 857-307-5422
Fax: 857-307-5476
Email: cscherzer@rics.bwh.harvard.edu

Index

Supplementary Figure 1. Flowchart of study participants……………………………………………………………..3

Supplementary Figure 2. Representative chromatograms of lipid extracts……………………………………………..4

Supplementary Table 1. Baseline characteristics of participants available for longitudinal analysis…………………5

Supplementary Table 2. The effect of *GBA* mutation status on the longitudinal change in CSF sphingolipid fractions……6

Supplementary Table 3. Baseline characteristics of idiopathic PD patients in each quartile of GlcCer/SM ratio measured at enrollment………………………………………………………………………………………………7

Supplementary Table 4. Association between baseline GlcCer/SM ratio and longitudinal MoCA scores in PD patients……….8

Supplementary Table 5. Baseline characteristics of GBA-PD patients in each quartile of GlcCer/SM ratio measured at enrollment………………………………………………………………………………………………9

Supplementary Table 6. The effect of the baseline GlcCer/SM ratio on the longitudinal change in MoCA scores in GBA-PD patients…………………………………………………………………………………………………..10

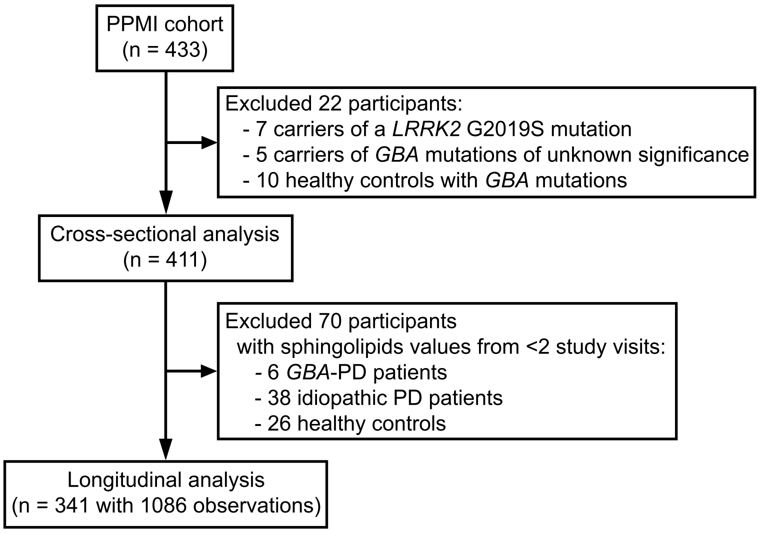

Supplementary Figure 1. Flowchart of study participants

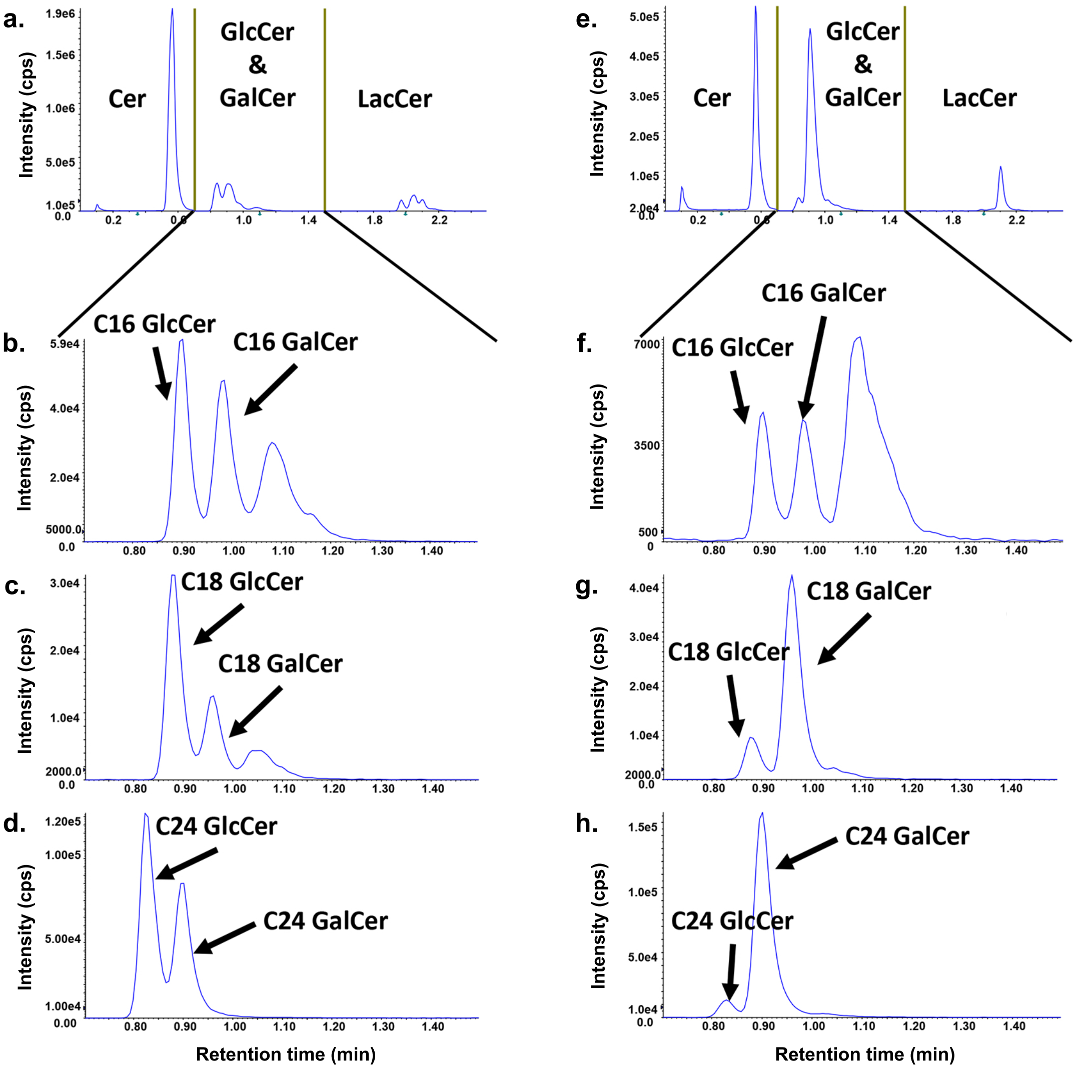

Supplementary Figure 2. Representative chromatograms of lipid extracts from standards in artificial CSF (a-d) and human CSF (e-h) with ceramides (Cer), glucosylceramides (GlcCer), and lactosylceramides (LacCer) detected by liquid chromatography-tandem mass spectrometry (LC-MS/MS). Sphingolipids were extracted from artificial CSF or human CSF as described in the methods. Chromatograms (a) and (e) display the total ion intensities for Cer, GlcCer/galactosylceramide (GalCer), and LacCer. Panels b through d and f through h are expanded views of the extracted ion chromatograms for GlcCer and GalCer in artificial and human CSF, respectively. Seven isoforms (C16, C18, C20, C22, C23, C24:1, and C24) of GlcCer and GalCer were analyzed and C16, C18, and C24 isoforms are shown as representatives. (a) Total ion chromatogram of lipid standard extract from artificial CSF. Standard mixtures (Cer, GlcCer, GalCer, and LacCer) were spiked into artificial CSF and extracted by liquid/liquid extraction. The resulting extract was analyzed by LC-MS/MS. Note that standards for GlcCer and GalCer are natural lipid mixture, both of which contain their own fatty acid distribution (See matreya.com for details). (b) Extracted ion chromatogram of C16 GlcCer and C16 GalCer in lipid standard extract from artificial CSF. (c) Extracted ion chromatogram of C18 GlcCer and C18 GalCer in lipid standard extract from artificial CSF. (d) Extracted ion chromatogram of C24 GlcCer and C24 GalCer in lipid standard extract from artificial CSF. (e) Total ion chromatogram of lipid extract from human CSF. Human CSF lipids were extracted by liquid/liquid extraction. The resulting extract was analyzed by LC-MS/MS. (f) Extracted ion chromatogram of C16 GlcCer and C16 GalCer in lipid extract from human CSF. (g) Extracted ion chromatogram of C18 GlcCer and C18 GalCer in lipid extract from human CSF. (h) Extracted ion chromatogram of C24 GlcCer and C24 GalCer in lipid extract from human CSF.

Supplementary Table 1. Baseline characteristics of participants available for longitudinal analysis

	HC (n=114)	Idiopathic PD (n=189)	*GBA*-PD (n=38)	*P* value
Age, y	63.0 (56.3, 70.0)	65.0 (58.0, 70.0)	58.0 (54.0, 64.0)	0.003*‡
Male, n (%)	77 (67.5%)	124 (65.6%)	23 (60.5%)	0.732
Age at onset, y	NA	65.0 (56.0, 69.0)	57.5 (54.0, 64.0)	0.001*
Disease duration, y	NA	1.0 (0.0, 1.0)	1.0 (0.0, 1.0)	0.936
MDS-UPDRS III	0.0 (0.0, 2.0)	21.0 (15.0, 29.0)	23.0 (15.3, 27.8)	< 0.001†‡
MoCA	28.0 (27.0, 29.0)	27.0 (26.0, 29.0)	28.0 (26.0, 29.0)	0.004†
Years of education	16.0 (15.0, 19.0)	16.0 (14.0, 18.0)	16.0 (14.3, 18.0)	0.106
BMI, kg/m^2	26.5 (24.1, 29.4)	26.4 (24.0, 30.1)	26.0 (24.3, 29.1)	0.844
Genotypes (n)	NA	NA	*GBA* risk variants (26)<br>: E326K (18), T369M (8)<br>Mild *GBA* mutations (6)<br>: N370S (6)<br>Severe *GBA* mutations (6)<br>: L444P (1), A456P (1), R463C (1), IVS2+1G>A (1), T369M/R120W (1), N370S/N370S (1)	

Values are median (interquartile range) unless otherwise stated.

[a] Kruskal-Wallis, Mann-Whitney, or χ^2 tests were used as appropriate.

**P* <0.05 *GBA*-PD vs idiopathic PD, †*P* <0.05 idiopathic PD vs HC, ‡ *P* <0.05 *GBA*-PD vs HC

BMI body mass index, *GBA-PD* Parkinson disease patients with a *GBA* mutation, *HC* healthy controls, *Idiopathic PD* Parkinson disease patients without *GBA* mutation, *MoCA* Montreal Cognitive Assessment, *MDS-UPDRS III* Movement Disorder Society-Unified Parkinson Disease Rating Scale part III.

Supplementary Table 2. The effect of *GBA* mutation status on the longitudinal change in CSF sphingolipid fractions

Glucosylceramide	β-coefficient	Standard error	*P* value
Age at baseline, y	0.004	0.002	0.006
Group (idiopathic PD)	0.012	0.042	0.764
Group (*GBA*-PD)	0.175	0.068	0.010
Time in study, y	0.010	0.014	0.476
Group (idiopathic PD) x time in study, y	0.019	0.018	0.292
Group (*GBA*-PD) x time in study, y	-0.042	0.027	0.120
**Sphingomyelin**	**β-coefficient**	**Standard error**	***P* value**
Group (idiopathic PD)	-0.009	0.052	0.858
Group (*GBA*-PD)	-0.228	0.083	0.006
Time in study, y	-0.019	0.020	0.328
Group (idiopathic PD) x time in study, y	-0.014	0.025	0.587
Group (*GBA*-PD) x time in study, y	0.060	0.038	0.111
**Ceramide**	**β-coefficient**	**Standard error**	***P* value**
Sex (male)	-0.080	0.022	0.0002
Group (idiopathic PD)	-0.002	0.028	0.932
Group (*GBA*-PD)	0.072	0.045	0.107
Time in study, y	0.010	0.013	0.411
Group (idiopathic PD) x time in study, y	-0.006	0.016	0.694
Group (*GBA*-PD) x time in study, y	-0.020	0.024	0.394

GBA-PD Parkinson disease patients with a *GBA* mutation, *Idiopathic PD* Parkinson disease patients without *GBA* mutation.

Supplementary Table 3. Baseline characteristics of idiopathic PD patients in each quartile of GlcCer/SM ratio measured at enrollment

Quartile of GlcCer/SM ratio<br>Range of GlcCer/SM ratio	Quartile 1 (n=48)<br>0.011 - 0.032	Quartile 2 (n=47)<br>0.008 - 0.011	Quartile 3 (n=47)<br>0.006 - 0.007	Quartile 4 (n=47)<br>0.001 - 0.006	*P* values
Age, y	66.00 (58.75, 70.25)	66.00 (60.00, 71.50)	64.00 (58.00, 71.00)	63.00 (55.50, 68.00)	0.492
Male, n (%)	32 (66.7%)	30 (63.8%)	30 (63.8%)	32 (68.1%)	0.963
Age at onset, y	65.50 (58.50, 69.25)	65.00 (58.50, 70.00)	63.00 (55.00, 70.00)	63.00 (55.00, 67.00)	0.545
Disease duration, y	1.00 (0.00, 1.25)	1.00 (0.00, 1.50)	1.00 (0.00, 1.00)	1.00 (0.00, 1.00)	0.674
MDS-UPDRS III	23.50 (16.00, 31.00)	20.00 (13.00, 23.50)	20.00 (15.00, 27.00)	20.00 (16.00, 26.00)	0.083
MoCA	27.00 (25.00, 28.00)	28.00 (26.50, 29.00)	27.00 (26.00, 28.00)	28.00 (26.00, 29.00)	0.067
Years of education	15.50 (14.00, 17.00)	16.00 (15.00, 18.00)	16.00 (14.00, 18.00)	16.00 (14.00, 18.00)	0.118
BMI, kg/m^2	26.23 (23.44, 30.32)	26.56 (24.41, 30.19)	25.69 (23.78, 30.10)	26.98 (24.43, 28.75)	0.791

Values are median (interquartile range) unless otherwise stated.

Kruskal-Wallis or χ^2 tests were used as appropriate.

BMI body mass index, *GlcCer/SM ratio* the ratio of glucosylceramide to sphingomyelin in the cerebrospinal fluid, *Idiopathic PD* = Parkinson disease patients without *GBA* mutation, *MoCA* Montreal Cognitive Assessment, *MDS-UPDRS III* Movement Disorder Society-Unified Parkinson Disease Rating Scale part III.

Supplementary Table 4. Association between baseline GlcCer/SM ratio and longitudinal MoCA scores in PD patients

	β-coefficient	Standard error	*P* value
Age at baseline, y	-0.05587	0.02154	0.011
Sex (male)	-1.26377	0.46624	0.008
Age at baseline x time in study, y	-0.02046	0.01112	0.069
Group (idiopathic PD patient in the highest quartile of baseline GlcCer/SM ratio)	-0.9056	0.43701	0.041
Time in study, y	1.00457	0.7085	0.159
Group (idiopathic PD patient in the highest quartile of baseline GlcCer/SM ratio) x time in study, y	-0.46126	0.22149	0.036

GlcCer/SM ratio the ratio of glucosylceramide to sphingomyelin in the cerebrospinal fluid, *Idiopathic PD* = Parkinson disease patients without *GBA* mutation.

Supplementary Table 5. Baseline characteristics of *GBA*-PD patients in each quartile of GlcCer/SM ratio measured at enrollment

Quartile of GlcCer/SM ratio<br>Range of GlcCer/SM ratio	Quartile 1 (n=10)<br>0.011 - 0.030	Quartile 2 (n=9)<br>0.009 - 0.011	Quartile 3 (n=9)<br>0.007 - 0.009	Quartile 4 (n=10)<br>0.003 - 0.007	*P* value
Age, y	65.0<br>(61.8, 69.8)	58.0<br>(55.0, 64.0)	55.0<br>(49.0, 59.0)	56.0<br>(51.8, 60.0)	0.029
Male, n (%)	5 (50.0%)	8 (88.9%)	4 (44.4%)	6 (60.0%)	0.215
Age at onset, y	64.0<br>(59.5, 68.5)	57.0<br>(54.0, 63.0)	54.0<br>(47.0, 59.0)	55.0<br>(51.8, 59.0)	0.037
Disease duration, y	1.0<br>(1.0, 1.8)	1.0<br>(0.0, 1.0)	0.0<br>(0.0, 1.0)	1.0<br>(0.0, 1.0)	0.299
MDS-UPDRS III	23.0<br>(16.3, 27.8)	25.0<br>(17.0, 30.0)	21.0<br>(16.0, 25.0)	17.0<br>(11.3, 25.3)	0.562
MoCA	26.0<br>(25.3, 29.0)	27.0<br>(26.0, 28.0)	29.0<br>(26.0, 29.0)	28.0<br>(25.5, 28.0)	0.587
Years of education	16.0<br>(14.3, 17.0)	16.0<br>(16.0, 18.0)	16.0<br>(15.0, 18.0)	16.0<br>(14.5, 16.0)	0.689
BMI, kg/m^2	26.8<br>(23.8, 28.8)	26.0<br>(24.1, 26.7)	24.7<br>(24.4, 27.0)	27.4<br>(24.6, 29.3)	0.851

Values are median (interquartile range) unless otherwise stated.

Kruskal-Wallis or χ^2 tests were used as appropriate.

BMI body mass index, *GlcCer/SM ratio* the ratio of glucosylceramide to sphingomyelin in the cerebrospinal fluid, *GBA-PD* = Parkinson disease patients with a *GBA* mutation, *MoCA* Montreal Cognitive Assessment, *MDS-UPDRS III* Movement Disorder Society-Unified Parkinson Disease Rating Scale part III.

Supplementary Table 6. The effect of the baseline GlcCer/SM ratio on the longitudinal change in MoCA scores in *GBA*-PD patients

	β-coefficient	Standard error	*P* value
Age at baseline, y	-0.837	0.346	0.024
Sex (male)	-1.394	0.494	0.010
Age at onset, y	0.754	0.338	0.036
Group (*GBA*-PD patient in the highest quartile of baseline GlcCer/SM ratio)	0.659	0.712	0.359
Time in study, y	0.238	0.306	0.448
Group (*GBA*-PD patient in the highest quartile of baseline GlcCer/SM ratio) x time in study, y	-1.102	0.466	0.029

GlcCer/SM ratio the ratio of glucosylceramide to sphingomyelin in the cerebrospinal fluid, *GBA-PD* = Parkinson disease patients with a *GBA* mutation.
