# Supplementary material for: Glucosylceramide in cerebrospinal fluid of patients with GBA-associated and idiopathic Parkinson’s disease enrolled in PPMI
Source: NPJ Parkinsons Dis. 2021 Nov 22;7:102. doi: 10.1038/s41531-021-00241-3 (PMC8608962; doi:10.1038/s41531-021-00241-3)
Supplement: Supplementary file 2 — Reporting Summary [file 41531_2021_241_MOESM2_ESM.pdf]

nature research

Corresponding author(s): Clemens R. Scherzer

Last updated by author(s): Sep 3, 2021

Reporting Summary

Nature Research wishes to improve the reproducibility of the work that we publish. This form provides structure for consistency and transparency in reporting. For further information on Nature Research policies, see our Editorial Policies and the Editorial Policy Checklist.

Statistics

For all statistical analyses, confirm that the following items are present in the figure legend, table legend, main text, or Methods section.

n/a	Confirmed	
☐	☒	The exact sample size (*n*) for each experimental group/condition, given as a discrete number and unit of measurement
☐	☒	A statement on whether measurements were taken from distinct samples or whether the same sample was measured repeatedly
☐	☒	The statistical test(s) used AND whether they are one- or two-sided<br>*Only common tests should be described solely by name; describe more complex techniques in the Methods section.*
☐	☒	A description of all covariates tested
☐	☒	A description of any assumptions or corrections, such as tests of normality and adjustment for multiple comparisons
☐	☒	A full description of the statistical parameters including central tendency (e.g. means) or other basic estimates (e.g. regression coefficient) AND variation (e.g. standard deviation) or associated estimates of uncertainty (e.g. confidence intervals)
☐	☒	For null hypothesis testing, the test statistic (e.g. *F*, *t*, *r*) with confidence intervals, effect sizes, degrees of freedom and *P* value noted<br>*Give P values as exact values whenever suitable.*
☒	☐	For Bayesian analysis, information on the choice of priors and Markov chain Monte Carlo settings
☒	☐	For hierarchical and complex designs, identification of the appropriate level for tests and full reporting of outcomes
☐	☒	Estimates of effect sizes (e.g. Cohen's *d*, Pearson's *r*), indicating how they were calculated

Our web collection on statistics for biologists contains articles on many of the points above.

Software and code

Policy information about availability of computer code

Data collection	No software was used for data collection.
Data analysis	A general linear mixed effects model analysis was performed using the lmer function in the lme4 package (version 1.1-21) in R (version 3.5.2).

For manuscripts utilizing custom algorithms or software that are central to the research but not yet described in published literature, software must be made available to editors and reviewers. We strongly encourage code deposition in a community repository (e.g. GitHub). See the Nature Research guidelines for submitting code & software for further information.

Data

Policy information about availability of data

All manuscripts must include a data availability statement. This statement should provide the following information, where applicable:

- Accession codes, unique identifiers, or web links for publicly available datasets
- A list of figures that have associated raw data
- A description of any restrictions on data availability

CSF samples, clinical and genetic data used in the preparation of this article were obtained from the Parkinson’s Progression Markers Initiative (PPMI) database (www.ppmi-info.org/data).

Field-specific reporting

Please select the one below that is the best fit for your research. If you are not sure, read the appropriate sections before making your selection.

☒ Life sciences ☐ Behavioural & social sciences ☐ Ecological, evolutionary & environmental sciences

For a reference copy of the document with all sections, see nature.com/documents/nr-reporting-summary-flat.pdf

Life sciences study design

All studies must disclose on these points even when the disclosure is negative.

Sample size	No sample size calculation was performed. Sample size was chosen based on the availability of data.
Data exclusions	Individuals with a known LRRK2 G2019S mutation were excluded for this analysis. GBA mutations of unknown significance, including G105R/G183E, I479L, R78C, R83C, were also excluded. Healthy controls with GBA mutations were excluded for this analysis. In the longitudinal analysis, participants with sphingolipids values from < 2 study visits were excluded.
Replication	Verifying the reproducibility is not applicable for this study due to the exploratory nature.
Randomization	In the PPMI study, PD patients were enrolled and longitudinally assessed; neither patients nor investigators were aware of genotype status; after completion of follow-up assessments, patients were assigned to carrier and non-carrier groups based on the genotypes.
Blinding	Participants and investigators were blinded to the genotype and sphingolipids data during the enrollment and the longitudinal follow-up.

Reporting for specific materials, systems and methods

We require information from authors about some types of materials, experimental systems and methods used in many studies. Here, indicate whether each material, system or method listed is relevant to your study. If you are not sure if a list item applies to your research, read the appropriate section before selecting a response.

Materials & experimental systems

n/a	Involved in the study	
☒	☐	Antibodies
☒	☐	Eukaryotic cell lines
☒	☐	Palaeontology and archaeology
☒	☐	Animals and other organisms
☐	☒	Human research participants
☒	☐	Clinical data
☒	☐	Dual use research of concern

Methods

n/a	Involved in the study	
☒	☐	ChIP-seq
☒	☐	Flow cytometry
☒	☐	MRI-based neuroimaging

Human research participants

Policy information about studies involving human research participants

Population characteristics	Characteristics of participants are described in Table 1.
Recruitment	All participants were recruited into PPMI cohort, a purpose-built biomarkers study with carefully standardized, highly compatible clinical and biospecimens collection methods. Participants were recruited and longitudinally assessed and both the participants and investigators were blinded to participants' genotype and sphingolipids data during the follow-up period. This study design is thought to be less vulnerable to recruitment and ascertainment bias than case-control studies.
Ethics oversight	The present analysis of de-identified data from PPMI was approved by the Institutional review board of Brigham and Women's Hospital. PPMI was approved by the ethics committees at each participating site, and written informed consent was obtained from all participants prior to inclusion in the study.

Note that full information on the approval of the study protocol must also be provided in the manuscript.
